# The effectiveness of different aerobic exercises to improve pain intensity and disability in chronic low back pain patients: a systematic review

**DOI:** 10.12688/f1000research.75440.2

**Published:** 2023-07-18

**Authors:** Shabbir Ahmed Sany, Maria Mitsi, Taukir Tanjim, Minhazur Rahman

**Affiliations:** 1 Department of Community Medicine, Faridpur Medical College, Faridpur, Dhaka, Bangladesh; 2 National Centre for Sport and Exercise Medicine, School of Sport,Exercise and Health Sciences, Loughborough University, Loughborough, Leicestershire, LE11 3TU, UK; 3 International Centre For Diarrhoeal Disease Research, Dhaka, Bangladesh

**Keywords:** Aerobic exercise, chronic low back pain, cycling, running, walking.

## Abstract

**Background:** Physical activity, including aerobic exercise, is highly recommended for chronic low back pain (CLBP) patients to improve pain intensity and functional disability.

**Objectives:** To assess the effectiveness of different aerobic exercises to reduce pain intensity and functional disability in patients with CLBP.

**Methods:** A computer-aided search was performed to find Randomised Controlled Trials (RCTs) that evaluated the effectiveness of different aerobic exercises in CLBP. Articles published between January 2007 to December 2020 were included in the review. Quality assessment using the PEDro scale, extraction of relevant information, and evaluation of outcomes were done by two reviewers independently.

**Results:** A total of 17 studies were included that involved 1146 participants. Outcomes suggested that aerobic exercise combined with other interventions was more effective than aerobic exercise alone. Aerobic exercise with higher frequency (≥ 5 days/week) and longer duration (≥ 12 weeks) were effective to gain clinically significant (≥ 30%) improvements. Environment and using pedometer did not seem to influence the outcomes.

**Conclusions:** Pain intensity and functional disability in CLBP patients can be minimized by prescribing aerobic exercise. However, to get better improvements, aerobic exercise should be done in combination with other interventions and at optimum frequency and duration. Further studies should emphasize examining the optimal doses and duration of different aerobic exercises.

## Introduction

Low back pain (LBP) is one of the leading causes of disability-related musculoskeletal conditions globally. ^1^^,^^2^ It is reported that 70-80% of the population suffer from LBP at some point in their lifetime. ^2^^,^^3^ 80-90% of those patients recover spontaneously from the acute phase of LBP within six weeks without taking any specific treatment. ^4^^–^^6^ However, the remaining 10-20% of patients develop chronic low back pain (CLBP), which is very difficult to treat and may lead to significant disability. ^5^^–^^7^ The National Health Service (NHS) spends more than £9 billion to provide CLBP patients treatment. ^8^ CLBP is now regarded as a significant public health problem globally, and the prevalence of CLBP has risen noticeably in the past decades. ^9^

Chronic low back pain (CLBP) is defined as pain, muscle tension, or stiffness located between the lower rib margins and above the lower gluteal folds that persists for more than 12 weeks (three months) with or without symptoms in the lower limbs. ^10^ Decreased physical activity is regarded as one of the main contributing factors to chronic musculoskeletal pain conditions, including CLBP. ^11^ Hence, the patients suffering from CLBP are encouraged to do regular exercise, and research showed that exercise was effective in preventing LBP by 35-45%. ^12^ Different clinical practice guidelines also recommended exercise as the first-choice treatment of CLBP. ^13^^–^^16^ Short-term and long-term improvements in pain and disability in CLBP patients can be achieved by doing exercises including aerobic exercise, flexibility training, stretching exercise, and resistance training. ^17^^–^^20^ However, there is still insufficient evidence regarding the best approach, intensity, and form of exercise program or physical activity that produces optimal outcomes for people with CLBP. ^19^^,^^21^

Aerobic exercise (AE) is one of the most recommended and widely used CLBP patients' interventions. ^22^^,^^23^ According to The American College of Sports Medicine (ACSM), AE is any structured physical activity that is rhythmic, uses large muscle groups of the body, and can be maintained continuously. ^24^^,^^25^ Walking, swimming, cycling, jogging, running, and hiking are typical AE examples. ^25^^,^^26^ Patients with CLBP can benefit from doing AE, as it increases the blood flow and nutrients supply to the soft tissue in the back, which facilitates the healing process. ^27^ Moreover, AE can significantly reduce pain intensity in CLBP patients by decreasing pain perception and muscle stiffness at the back. ^18^^,^^28^^,^^29^

Recently, some systematic reviews and meta-analyses, including by Vanti *et al.,* ^30^ Sitthipornvorakul *et al.,* ^31^ and Lawford *et al.,* ^32^ showed that walking exercise was as effective as other exercise and non-pharmacological interventions. Nevertheless, they evaluated only walking exercise; therefore, other forms of aerobic exercise were missed. To our knowledge, only two reviews have been conducted so far that assessed the effectiveness of different AE in CLBP patients. ^23^^,^^33^ However, neither of these reviews included a study published after 2013. Therefore, we carried out this systematic review to evaluate the recently published articles and provide up-to-date information. This review aimed to evaluate different AE's effectiveness in improving pain intensity and disability in CLBP patients.

## Methods

This review was conducted following the Preferred Reporting Items for Systematic Reviews and Meta-Analyses (PRISMA) guidelines. ^34^

### Data sources and searches

Different databases were searched, including PubMed, CINAHL, PEDro, MEDLINE, and SPORTDiscus, to identify the relevant studies. We searched articles published between January 2007 to December 2020 as we focused on evaluating the recently published articles. The following keywords were used independently and in combination: low back pain, backache, aerobic exercise, walking, treadmill walking, cycling. A brief description of the of the search query is shown in the Appendix 1. The first and second authors examined titles, abstracts, and complete articles of potentially relevant papers independently to determine eligibility. Any disagreements on eligibility were scrutinized by the third and fourth authors and were resolved through discussion.

**Appendix 1.** Search strategies. Last searched December 2020.

PubMed	The following keywords were used independently and in combination: low back pain, backache, aerobic exercise, walking, treadmill walking, cycling.
CINAHL	back pain; low back pain; backache; exercise; (MH “Lumbar Vertebrae”); (MH “Exercise+”); (MH “Physical Therapy+”); (MH “Aerobic Exercise+”); (MH “walking+”); (MH “Cycling+”).
PEDro	Therapy: strength training, education, stretching, mobilization, manipulation, massage, fitness training. Problem: Pain. Body part: Lumbar spine, sacroiliac joint or pelvis. Topic: Chronic pain.Method: Blank.
MEDLINE	randomized controlled trial.pt.; controlled clinical trial.pt.; random*.ti,ab.; trial.ab,ti.; groups.ab,ti.; (backache or back pain).tw,kf.; exp Back Pain/; (lumb* adj pain).tw,kf.; exp low back pain/; exp Exercise/; exp Exercise Therapy/; (walking or treadmill walking).tw,kf; exp Aerobic Exercise/
SportDiscus	Clinical trials; random*; double blind; random allocation; controlled clinical trial; back pain; backache; lumb*pain; exercise; DE “aerobic exercise”; DE “cycling”; DE “walking”.

### Criteria for considering studies for this review

Types of studies:

In this systematic review, we included randomized controlled trials (RCTs) that evaluated the effectiveness of AE with or without other interventions in at least one group. We only considered the studies that were published in the English language. The study that investigated patients with chronic low back pain and acute and/or subacute LBP together was also excluded. Papers published before 2007 and other than the English language were excluded.

Types of participants:

We included the studies involving participants aged ≥18 years with low back pain for a minimum of 3 months (≥12 weeks). Studies that involved patients with a history of acute or subacute low back pain, cauda equine syndrome, inflammatory or tumoral back conditions, osteoporosis of the spine and pregnancy, and surgery in the lumbosacral region, spinal fracture, and dislocation were excluded in this systematic review.

Types of outcome measures:

Studies that evaluated at least pain intensity or functional disability as the outcome with or without other measurements were included in this systematic review.

Details of inclusion and exclusion criteria are shown in Table 1.

**Table 1.** Summary of inclusion and exclusion criteria.

	Inclusion criteria	Exclusion criteria
Types of studies	• Used a randomized controlled trial design. • Examined aerobic exercise with or without other intervention in at least one group. • Papers published between January 2007 to December 2020.	• The study that investigated patients with chronic low back pain and acute and/or subacute LBP together. • Papers published other than the English language.
Types of participants	• Aged ≥ 18 years • Patients with low back pain for a minimum of 3 months (≥12 weeks).	• Patients with acute or subacute low back pain. • Patients with cauda equine syndrome. • Patients with inflammatory or tumoral back conditions. • Patients with osteoporosis of spine and pregnancy. • Patients with a history of surgery in the lumbosacral region, spinal fracture, and dislocation.
Types of outcome measures	• Studies that evaluated at least pain intensity or functional disability as the outcome with or without other measurements.	

### Assessment of risk of bias and quality of studies

The Physiotherapy Evidence Database (PEDro) scale was employed to assess the methodological quality and risk of bias of included studies. PEDro scale is regarded as a valid and reliable risk of bias tool. ^35^^,^^36^ PEDro scale has 11 components or items including eligibility criteria, random allocation, concealed allocation, baseline similarity, blind subjects, blind therapists, blind assessors, sufficient follow up (85% follow up for at least one key outcome), intention-to-treat analysis, between-group statistic comparison (for at least one key outcome), and point estimates and variability (for at least one key outcome). ^37^ Eight items (item 2-9) are used to evaluate the risk of bias and last two items (10 and 11) are related to statistical reporting. ^36^ The first item which is eligibility criteria is not counted in the total score as it is related to external validity. Hence, PEDro score ranges between 0 and 10 points, where the article with higher score regarded as better article in terms of risk of bias and statistical reporting. Any study with a score between 6 and 10, score with 4 or 5, and score ≤ 3 is considered good quality, fair quality, and poor quality study, respectively. ^38^^,^^39^ However, it is impossible to blind therapists and all subjects in clinical trials because of ethical standards. Hence any study with a score of 8/10 is regarded as low risk of bias. ^39^ PEDro scoring was done using the PEDro scale by the first two authors independently, and the other two authors resolved any discrepancy through discussion.

### Types of outcome measures

In this systematic review, we evaluated the effectiveness of different AE in improving pain intensity and disability in CLBP patients. Therefore, we reported the following outcomes:

• Pain intensity: Pain intensity measured by a pain scale, including Visual Analogue Scale (VAS), Numerical Pain Rating Scale (NRS), and McGill Pain Questionnaire (MPQ).
• Functional disability: Functional disability evaluated by using Oswestry Low Back Pain Disability Index (ODI, 0-100), Oswestry Low Back Pain Disability Index (ODI, 10-60), Roland and Morris Disability Questionnaire (RMDQ), and Aberdeen Low Back Pain Disability Scale (ALBPS).

Outcome measurements of different time points were included in the analysis to evaluate the treatment effect described below.

### Data synthesis and measurement of treatment effect

Required data were extracted from studies by using a data extraction form. The first two reviewers extracted relevant information on sample size and subject characteristics; type, frequency, intensity, and duration of interventions; instruments used to assess the outcomes; and outcomes of pain intensity and functional disability. The third and fourth authors further evaluated the extracted data, and any disagreements were resolved through discussion. The outcomes were continuous variables, and treatment effects were reported as mean differences and mean percentage changes. Mean percentage changes ≥ 30% were regarded as minimal clinically significant differences (MCID) described by the previous studies. ^40^^–^^42^ We did not perform the meta-analysis due to the heterogenicity of participants, intervention, and outcome measures.

## Results

### Data search

**Figure 1.**
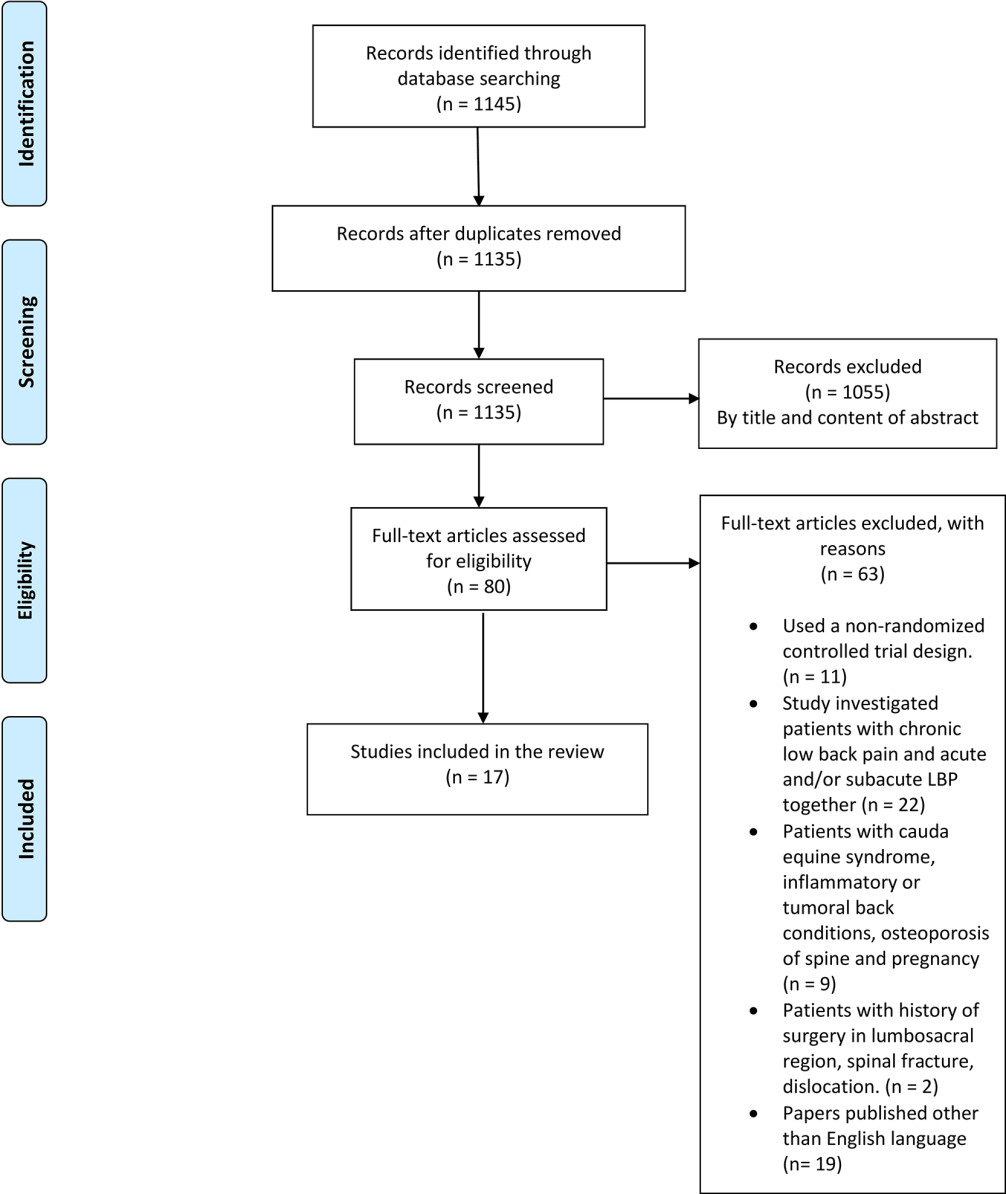
PRISMA flow diagram (Study selection process).

After searching databases, a total of 1,145 studies were identified. After removing duplicates, 1135 articles were screened by title and content of the abstract. After that screening, 1055 articles were excluded, and the remaining 80 articles were evaluated for eligibility. Finally, a total of 17 studies met all inclusion criteria and were included for this review. A PRISMA flow diagram of the study selection process is shown in Figure 1.

### Study characteristics

All included studies were RCTs, involved a total of 1146 patients. The number of participants ranging from 14 ^43^ to 246, ^44^ and patients were >18 years. The duration of included studies' intervention was between 4 weeks ^45^ and 12 months. ^46^ Among these, eleven studies followed up for short term (<3 months or 12 weeks), ^45^^,^^47^^–^^56^ eleven studies followed up for intermediate-term (3 months to <12 months) ^43^^,^^44^^,^^46^^–^^48^^,^^52^^,^^53^^,^^56^^–^^59^ and two studies followed up for long term (≥12 months). ^46^^,^^55^ Studies examined the effectiveness of different types of AE. Eleven studies evaluated the effectiveness of different forms of AE alone ^43^^–^^47^^,^^49^^,^^54^^,^^56^^–^^58^ while seven studies examined AE in conjunction with other intervention including stabilization exercise, ^47^ back school program, ^50^ rehabilitation program, ^51^ group exercise class, ^52^ traditional physiotherapy ^55^ and home exercise. ^48^

As an intervention, ten studies used walking exercise ^45^^–^^49^^,^^51^^,^^53^^,^^54^^,^^58^; three studies used stationary cycling exercise ^45^^,^^50^^,^^52^; one study used treadmill running exercise ^57^; one study used both walking and running exercise ^43^; one study used combined treadmill walking, stair climbing and stationary cycling exercise ^59^; one study used walking and jogging exercise with elliptical trainer ^56^; one study used individually designed and supervised aerobic exercise. ^55^

In this review, we focused on the improvement in pain intensity and functional disability. To measure pain intensity, nine studies used the Visual Analogic Scale (VAS), ^43^^,^^47^^–^^49^^,^^51^^,^^52^^,^^55^^,^^56^^,^^59^ six studies used Numerical Pain Rating Scale (NRS), ^44^^–^^46^^,^^50^^,^^53^^,^^58^ one study used McGill Pain Questionnaire (MPQ) ^57^ and one study did not measure pain intensity. ^54^ Functional disability was evaluated by using Oswestry Low Back Pain Disability Index (ODI, 0-100) in eleven studies, ^43^^–^^45^^,^^47^^,^^49^^,^^52^^–^^54^^,^^56^^,^^58^^,^^59^ Oswestry Low Back Pain Disability Index (ODI,10-60) in one study, ^51^ Roland and Morris Disability Questionnaire (RMDQ) in four studies ^46^^,^^48^^,^^50^^,^^57^ and Aberdeen Low Back Pain Disability Scale (ALBPS) in one study. ^55^

### Risk of bias and quality assessment of studies

Quality assessment of included studies using the PEDro scale is shown in Table 2, which demonstrated that the range of the scores was between 4/10 and 8/10 (mean 6.35 ± 1.46). Two studies scored the minimum (4/10), ^45^^,^^51^ while five studies reached the best possible score (8/10). ^49^^,^^52^^–^^54^^,^^58^ Eleven studies obtained the threshold score (6/10) to be considered a good quality study. ^43^^,^^44^^,^^46^^,^^47^^,^^49^^,^^52^^–^^55^^,^^58^^,^^59^ Studies that scored the lowest had a lack of concealed allocation, blind assessors, adequate follow-up, and intention-to-treat analysis. ^45^^,^^51^

**Table 2.** Quality assessment of included studies using PEDro scale.

Paper name and reference	Eligibility criteria	Random allocation	Concealed allocation	Baseline comparability	Blind subjects	Blind therapists	Blind assessors	Adequate follow-up	Intention-to-treat analysis	Between group comparisons	Point estimates and variability	Total score
Kanitz *et al.* ^43^	Y	Y	Y	Y	N	N	Y	Y	N	Y	Y	7/10
Suh *et al.* ^47^	Y	Y	Y	Y	N	N	Y	N	N	Y	Y	6/10
Barni *et al.* ^50^	Y	Y	Y	Y	N	N	N	N	N	Y	Y	5/10
Bello and Adeniyi ^49^	Y	Y	Y	Y	N	N	Y	Y	Y	Y	Y	8/10
Chulliyil *et al.* ^45^	Y	Y	N	Y	N	N	N	N	N	Y	Y	4/10
Cho *et al.* ^51^	Y	Y	N	Y	N	N	N	N	N	Y	Y	4/10
Hurley *et al.* ^44^	Y	Y	N	Y	N	N	Y	Y	Y	Y	Y	7/10
Eadie *et al.* ^58^	Y	Y	Y	Y	N	N	Y	Y	Y	Y	Y	8/10
Krein *et al.* ^46^	Y	Y	Y	Y	N	N	N	Y	Y	Y	Y	7/10
Marshall *et al.* ^52^	Y	Y	Y	Y	N	N	Y	Y	Y	Y	Y	8/10
McDonough *et al.* ^53^	Y	Y	Y	Y	N	N	Y	Y	Y	Y	Y	8/10
Shnayderman and Katz-Leurer ^54^	Y	Y	Y	Y	N	N	Y	Y	Y	Y	Y	8/10
Chan *et al.* ^55^	Y	Y	Y	Y	N	N	N	Y	Y	Y	Y	7/10
Murtezani *et al.* ^59^	Y	Y	Y	Y	N	N	N	Y	Y	N	Y	6/10
Kell and Asmundson ^56^	Y	Y	N	Y	N	N	N	Y	N	Y	Y	5/10
Koldas *et al.* ^48^	Y	Y	N	Y	N	N	N	Y	N	Y	Y	5/10
Chatzitheodorou *et al.* ^57^	Y	Y	N	Y	N	N	N	Y	N	Y	Y	5/10

### Effectiveness of different aerobic exercises to improve CLBP

*Walking alone exercise*

Bello *et al.* ^50^ compared walking exercise alone to lumbar stabilization exercise by involving a total of 50 patients who were divided into walking exercise group (WG) and lumbar stabilization exercise group (LSG). WG received walking exercise on the treadmill at an intensity of 65-80% HRR, while LSG received 30 mins of lumbar stabilization exercises following the McGill protocol for eight weeks (three times/week). ^50^ After intervention, both groups showed improvement in pain (WG vs LSG = 32.8% vs 59.4%) and disability (WG vs LSG = 14.4% vs 48.9%), while LSG demonstrated better outcomes. ^50^

In another study, Shnayderman and Katz-Leurer ^54^ evaluated the effectiveness of walking exercise (WG) against specific low back strengthening exercises (SG) by recruiting 52 patients. WG received 40 minutes of walking exercise on the treadmill at 50% heart rate reserve. ^54^ Both groups received exercises two days a week for six weeks. ^54^ Outcomes revealed that both groups showed significant improvements in disability without significant differences between groups where ODI scores were reduced by 34.3% in WG and 30.6% in SG. ^54^

Both studies' significant limitations included lack of a control group, no long-term follow-up, and a short intervention period. Conversely, Hurley *et al.* ^44^ evaluated walking exercise's effectiveness with a larger sample size and long-term follow-up. 246 patients aged 18-65 were equally divided into three groups. The first group (WG) received supervised walking exercise for a minimum of 10-minutes to 30 minutes walk/day at 40-60% HRR, at least four times/week for seven weeks. ^44^ The second group (ECG) trained with group exercise class (Back to fitness program, a one-hour long class per week for eight weeks) and exercise including warm-up and stretching. The third group (UG) received usual physiotherapy. ^44^ Results showed that pain and disability improved in all three groups. ^44^ Authors reported that 48% in WG, 45% in ECG, and 31% in UG participants achieved minimal clinically significant difference (MCID) in the ODI score. ^44^ Whereas 44%, 29%, and 37% of WG, ECG, and UG participants reached MCID in the NRS score. ^44^ Authors also reported that the walking program had the greatest adherence and the lowest costs. ^44^ A significant limitation of this study was that a total of 40 therapists were involved in this study to train the patients; hence therapist effects could influence the outcomes.

*Walking exercise in conjunction with other intervention*

Cho *et al.* ^51^ studied whether treadmill walking exercise combined with a low back pain rehabilitation program helped reduce pain and disability in CLBP patients. Twenty men were equally divided into an experimental group (EG) and a control group (CG). ^51^ EG received treadmill walking exercise without a slope at 3-3.5 km/h, for 30 minutess and low back pain rehabilitation program; whereas, CG received only a low back pain rehabilitation program. ^51^ Both groups received 30 minutes long low back pain rehabilitation program, three days/week, and the duration of intervention was eight weeks. ^51^ After the intervention, both groups showed improvement in pain (VAS) and disability (ODI) scores without any significant difference between groups. ^51^ In EG, VAS and ODI scores were reduced by 46.1% (vs 43.5% in CG) and 21.5% (vs 12.7% in CG) respectively. Overall, additional treadmill exercise did not provide additional improvements. ^51^ The small sample size was a major drawback of this study.

Koldas *et al.* ^48^ experimented with a larger sample of sixty patients to examine combined walking exercise and home exercise effectiveness. Twenty patients (AHE) received 40-50 minutes of exercise on a treadmill at 65-70% HRR, three times/week with home exercise. ^48^ At the same time, the remaining 40 patients were assigned to receive either physical therapy (PT) or home exercise only (HE). ^48^ Home exercises included basic flexion, extension, mobilization, and stretching, and the patients were asked to perform the exercise once a day with 15-20 repetitions. ^48^ All groups received their specific exercises for six weeks. ^48^ Results showed that pain reduced significantly in all three groups after the treatment (AHE vs. PT vs. HE: 39.6% vs. 36.5% vs. 28.6%) and at one-month follow-up (AHE vs. PT vs. HE: 38.8% vs. 53% vs. 40%). ^48^ Disability was improved significantly in AHE and PT at both post-intervention (25.2% vs. 25.2%) and follow-up (22.7% vs. 30.3%), while in HE, it was negligible. ^48^

In another study, Suh *et al.* ^47^ utilized 48 patients aged > 20 years to compare walking exercise alone (WE) to three different interventions, including combined walking and stabilization exercise (SWE), flexibility exercise (FE), and only stabilization exercise (SE). The WE group received 30 mins of fast walking exercise on flat ground with abdominal bracing, whereas the SWE group trained with 30 minutes of walking exercise and 30 minutes of stabilization exercise. ^47^ Outcomes were measured at baseline, within two weeks after intervention and six weeks after the intervention. ^47^ Results indicated that pain intensity decreased in all four groups both during activity (FE vs WE vs SE vs SWE: 45.26% vs 38.7% vs 48.19% vs 44.06%) and at rest (FE vs WE vs SE vs SWE: 33.61% vs 18.25% vs 35.33% vs 38.9%) after the intervention. ^47^ Further assessment at six weeks after intervention showed that all groups retained enhancement in pain scores. ^47^ Disability evaluation demonstrated that after the intervention, the ODI score was decreased by 19.93% in WE; while in FE, SE and SWE, it was 16.05%, 19.43%, and 18.09%, respectively. ^47^ The frequency of exercise in SE and WE increased significantly after the intervention. However, the SWE group showed the opposite trend, which demonstrated poor adherence to exercise, and it was difficult for the participants of the SWE group to perform 60 minutes of exercise. ^47^

*Pedometer-driven walking exercise*

Eadie *et al.* ^58^ experimented on 60 patients aged 18-70 years by distributing them into three groups (WG, SG, PG). WG received walking exercise for 30 minutes at moderate intensity, five days/week, and they were asked to wear a pedometer during walking to record the progress. ^58^ SG Received a supervised exercise class (back to fitness program) once per week, whereas PG received usual physiotherapy. ^58^ The total intervention duration was eight weeks, and outcomes were measured at three months and six months. ^58^ Pain score evaluation showed that both WG (12.1%) and SG (12.9%) gained similar improvements, whereas PG (32%) obtained more significant improvements. ^58^ However, unlike the other two groups, WG (-1.79%) failed to retain the improvements at six months. ^58^ Moreover, the smallest improvement in disability also was in WG (9.4%) compared to SG (22%) and PG (27.3%). ^58^ The authors reported small sample size and a high drop-out rate during the follow-up period could impact the outcomes. ^58^

Besides, McDonough *et al.* ^53^ examined 57 patients aged between 43 and 53 years. The experimental group (EG) received combined pedometer-driven walking exercise and education, whereas the control group (CG) received only education or advice. Participants were familiarized with wearing a pedometer, and they were asked to record their daily steps in a walking diary. ^53^ The intervention duration was nine weeks, and measurements were done after intervention and six months after randomization. ^53^ Results indicated pain intensity improved in both groups; however, EG showed greater improvement (16.7% vs. 15.2% at nine weeks and 29.6% vs. 10.9% at six months). ^53^ EG showed a better outcome in disability (17.2% vs 3.3% at nine weeks and 25.7% vs 5.5% at six months). ^53^ However, the sample size was relatively small, which was a weakness of this study.

In contrast, Krein *et al.* ^46^ experimented with a larger sample size and long-term follow-up to examine whether additional support affected patients' improvement. They examined 229 patients by separating them into two groups: experimental group (EG) and usual care group or control group (CG). ^46^ EG received an uploading pedometer and additional support, access to a website that provided information about walking goal progress, and patients received feedback, motivational and informational messages. ^46^ In contrast, CG received an uploading pedometer but did not receive any walking goal and did not access the website. ^46^ This study's duration was 12 months, and outcome measurements were done at baseline, six months, and 12 months. ^46^ Results demonstrated pain intensity improved in both groups at six months (EG vs CG = 21.7% vs 14.8%) and 12 months (EG vs CG = 10% vs 8.2%), where improvements were greater at six months. ^46^ Disability improvement was also greater in EG at six months (20.9% vs. 6.1%). ^46^ Patients were recruited from one medical center, which was indicated as a limitation of this study. ^46^

*Stationary cycling exercise*

Barni *et al.* ^50^ evaluated the effectiveness of a combined back school program and stationary cycling exercise by recruiting 22 patients. Patients were assigned to either the experimental group (EG) who received exercise on the stationary bike at 65% HRR and back school program; or the control group (CG) who received only the back school program. ^50^ The interventions' total duration was five weeks (90 minutes session, two sessions/week). ^50^ Post-intervention measurements showed a greater reduction in NRS and RMDQ index in the experimental group than in the control group (NRS = 27% vs 13.14% and RMDQ = 25.58% vs 10.3%). ^50^ This study's major limitations were lack of long-term follow-up, short duration of intervention, and a small number of participants.

Marshall *et al.* ^52^ experimented with a relatively larger sample size (64 patients) and a longer duration to observe whether stationary cycling exercise combined with exercise class (CEG) was more effective than specific trunk exercise conjunction with exercise class (SEG). Both groups received their specific exercise for 35-40 minutes and 50-60 minutes of exercise classes (three sessions/week) for eight weeks. ^52^ Outcomes were recorded at baseline, post-intervention, and six months from the start of the intervention. ^52^ Results showed that pain decreased in both groups (SEG vs CEG = after intervention: 52.8% vs 17.8%, at six months: 44.4% vs 26.7%), where SEG showed better improvements. ^52^ 56% of SEG and 50% of CEG participants showed clinically relevant changes (≥ 30%) after intervention. ^52^ Disability was significantly lower in SEG compared to CEG after the intervention (40.9% vs. 16.3%), and 66% of SEG and 44% of CEG participants demonstrated clinically significant change (≥30%) in ODI score. ^52^ The authors concluded that both exercises effectively improved pain and disability without significant differences between groups. ^52^ Although trunk exercise showed better improvements than stationary cycling immediately after the intervention, long-term follow-up outcomes were similar. ^52^

Chulliyil *et al.* ^45^ examined whether stationary cycling exercise is superior to treadmill walking to improve CLBP. A total of 30 patients aged 18-30 years were divided into two groups. ^45^ One group (TG) received AE by treadmill walking, while another group (SCG) received AE by stationary cycling. ^45^ Both groups received AE at moderate intensity (13-14 RPE) for 10-20 minutes for four weeks (five days/week). ^45^ Post-intervention measurements demonstrated that both groups showed significant improvements in all measurements without any significant differences between groups (TG vs SCG = NRS at rest: 80.1% vs 69.3%, NRS on activity: 55.4% vs 45.3%, ODI: 53.8% vs 49.8%). ^45^ However, the very short duration of the study and the absence of long-term follow-up were the major drawbacks of this study.

*Treadmill running exercise*

Chatzitheodorou *et al.* ^57^ recruited 20 patients to examine the effectiveness of running exercise to improve CLBP. Ten patients in the experimental group (EG) received high-intensity AE by running on the treadmill at 60%-85% of HRR, 30-50 minutes session, three sessions/week. ^57^ Whereas the remaining ten patients in the control group (CG) received passive modalities (45 minute session) without any physical activity. ^57^ After intervention (12 weeks) EG showed significantly better improvement in pain (40.1% vs. 0.6%) and disability (30.4% vs. 0.7%) compared to CG. ^57^

*Combination of different aerobic exercises*

Murtezani *et al.* ^59^ examined 101 patients with CLBP by assigning them either to the experimental group (EG) or the Control group (CG). EG received high-intensity AE, including treadmill walking, stair climbing, and stationary bicycling at 50%-85% HRR, 30-50 mins sessions, three sessions/week for 12 weeks. ^59^ In contrast, CG received passive modalities (45 min session, three times/week). ^59^ Results showed significant improvements in all parameters in EG (pain 66.7% and disability 49%), while the improvements in CG were non-significant (pain 1.6%, disability 0.3%). ^59^

*Individually designed and supervised aerobic exercise*

Chan *et al.* ^55^ utilized 46 patients who were included either in the experimental group (EG) or control group (CG). EG received conventional physiotherapy and 20 minutes of aerobic training at 40%-60% HRR, gradually progressed up to 85% at a 5% increment each weak for eight weeks (three sessions per week). ^55^ Subjects in EG were also asked to perform a minimum of one additional training per week at home. ^55^ Participants selected the type of that training according to their preference, including treadmill walking or running, stepping, and cycling. ^55^ Subjects in the control group received only conventional physiotherapy for eight weeks. ^55^ Post-intervention measurements indicated significant improvements in pain and disability in both groups without any significant differences between groups. ^55^ EG attained clinically significant improvements (≥ 30%) in VAS and ALBPS scores at all time points. ^55^ The short duration of intervention and relatively small sample size are major limitations of this study. ^55^ In addition, the authors reported poor baseline fitness level of patients, which could be a factor to influence the outcomes. ^55^

*Influence of the environment*

Kanitz *et al.* ^43^ experimented on 14 patients to see the impact of the environment on AE outcomes. Participants were randomly allocated into two groups and received 35 minutes of walking/running exercise at moderate intensity (85-95% HRvt2) for 12 weeks (two times/week) either on land (LG) or in water (AG). ^43^ Outcomes showed improvements in pain and disability score in both groups without any difference between groups (LG vs. AG = pain: 66.67% vs. 47.27% and disability: 40.59% vs. 48%). ^43^ However, small sample size and absence of long-term follow-up were reported as weaknesses of this study. ^43^

*Periodized progressive overload training*

Kell and Asmundson ^56^ carried out an experiment to observe periodized progressive overload training effectiveness. A total of 27 patients with CLBP were equally divided into three groups (AT, RT, and CG). ^56^ AT received periodized progressive overload aerobic training (elliptical trainer and treadmill walking and jogging. 20-35 min session, three sessions/week), while RT received periodized resistance training. ^56^ Patients in the control group (CG) maintained regular activity. ^56^ The intervention's total duration was 14 weeks, consisting of two phases (seven weeks per phase). ^56^ Outcomes were measured after completing each phase (at eight weeks and 16 weeks). ^56^ Results showed significant improvement in pain (27.8% at eight weeks and 38.9% at 16 weeks) and disability (30.2% at 8 weeks and 40.1% at 16 weeks) in RT. In contrast, AT demonstrated improvement only in cardiorespiratory performance (VO2max), body fat, and body mass. ^56^ However, an experiment with a larger sample size is needed to conclude the efficacy of periodized progressive overload aerobic training on CLBP patients.

The summary of interventions, measurements, outcomes, and main limitations of the included studies are shown in Table 3 and Table 4.

**Table 3.** Overview of included studies that evaluated the effectiveness of aerobic exercise alone intervention.

Study and subjects	Intervention	Measurements	Key outcomes	Mean change in percentage (absolute value change) pain	Mean change in percentage (absolute value change) disability	Key limitations
Kanitz *et al.* ^43^ 14 patients, Aged: 30-50 years	• Group 1 (LG): Received aerobic exercise on land. • Group 2 (AG): Received aerobic exercise in deep water. • Aerobic exercise included walking/running at moderate intensity (85-95% HRvt2) for 35 mins. • Duration: 12 weeks (Two times a week).	• Pain (VAS) • Disability (ODI) • Measurements were done at baseline and after the intervention	✓ Improvements in pain and disability score were observed in both groups without any difference between groups.	Scale: VAS (100 mm) After intervention (12 weeks): LG: −66.67% (−3.8) AG: −47.27% (−2.6)	Scale: ODI (0-100) After intervention (12 weeks): LG: −40.59% (−4.1) AG: −48% (−4.8)	▪ Small sample size. ▪ Low frequency of training. ▪ No long-term follow-up.
Bello and Adeniyi ^49^ 50 patients, Aged: 18-60 years	• Group 1 (LSG): Received lumbar stabilization exercises following the McGill protocol (30 minutes session). • Group 2 (WG): Received treadmill walking exercise (at 65%-80% HRR). • Duration: 8 weeks (three times a week).	• Pain intensity (VAS). • Functional disability (ODI). • Measurements were done at baseline and after the intervention.	✓ Significant improvements in pain intensity and disability in both groups were observed. ✓ LSG showed significantly greater improvements in all parameters than WG.	Scale: VAS (0-10 cm) After intervention (8 weeks): LSG: −59.4% (−3.8) WG: −32.8% (−2.2)	Scale: ODI (0-100) After intervention (8 weeks): LSG: −48.9% (−23.1) WG: −14.4% (−6.7)	▪ EMG activity of muscles could not be measured by using needle insertion. ▪ No long-term follow-up. ▪ Short duration of intervention.
Chulliyil *et al.* ^45^ 30 patients, Aged: 18-45 years	• Group 1 (TG): Received aerobic exercise by treadmill walking. • Group 2 (SCG): Received aerobic exercise by stationary cycling. • Aerobic exercise was at moderate intensity (13-14 RPE) for 10-20 mins. • Duration: 4 weeks (5 days/week).	• Pain intensity at rest and on activity (NRS) • Disability (modified ODI) • Measurements were done at baseline and after the intervention.	✓ Both groups showed significant improvements in all measurements without any significant differences between groups.	Scale: NRS (0-10 point) After intervention (4 weeks): At rest: TG: −80.1% (−2.94) SCG: −69.3% (−2.86) On activity: TG: −55.4% (−3.73) SCG: −45.3% (−3.2)	Scale: ODI (0-100) After intervention (4 weeks): TG: −53.8% (−24.53) SCG: −49.8% (−26.4)	▪ Very short duration of intervention. ▪ Man and woman participants were not the same in number. ▪ No follow-up was done.
Hurley *et al.* ^44^ 246 patients, Aged: 18-65 years	• Group 1 (WG): Received supervised walking exercise (minimum 10 min to 30 min walk/day at 40-60% HRR, for at least 4 days per week for 7 weeks. • Group 2 (ECG): Received group exercise class (Back to fitness program, a one-hour long class per week for 8 weeks.) and exercise including warm-up and stretching. • Group 3 (UG): Usual physiotherapy (education, exercise therapy, and manipulative therapy). • Duration: 8 weeks	• Pain (NRS 0-10) • Functional disability (ODI) • Measurements were done at baseline and at 3, 6, and 12 months after randomization.	✓ Significant improvements were observed in all three groups, with no significant differences between groups at all time points. ✓ No difference in the efficacy of all three programs. ✓ WG had the greatest adherence ✓ WG had the lowest costs.	Scale: NRS (0-10) At 3 months: WG: −17.8% (−0.97) ECG: −7.6% (−0.43) UG: −19.3% (−1.16) At 6 months: WG: −20.9% (−1.14) ECG: −9.4% (−0.53) UG: −15.8% (−0.95) At 12 months: WG: −21.4% (−1.17) ECG: −11% (−0.62) UG: −18.1% (−1.09)	Scale: ODI (0-100) At 3 months: WG: −12.8% (−4.45) ECG: −13% (−4.95) UG: −18% (−5.98) At 6 months: WG: −19.8% (−6.89) ECG: −15.5% (−5.91) UG: −15.3% (−5.09) At 12 months: WG: −18.7% (−6.51) ECG: −21% (−8) UG: −14.8% (−4.91)	▪ Lack of blinding of therapists and patients. ▪ Treating therapists were high in number.
Eadie *et al.* ^58^ 60 patients, Aged: 18-70 years	• Group 1 (WG): Received walking exercise (30 mins at moderate intensity, 5 days per week). (Pedometer was used to record the progress) • Group 2 (SG): Received supervised exercise class (back to fitness program, once per week). • Group 3 (PG): Received usual physiotherapy (advice, manual therapy, and exercise) • Duration: 8 weeks.	• Pain (NRS) • Disability (ODI) • Measurements were done baseline, 3 months, and 6 months.	✓ Greater pain intensity and disability improvements were observed in PG than WG and SG in all time points.	Scale: NRS (0-10) At 3 months: WG: −12.1% (−0.68) SG: −12.9% (−0.68) PG: −32% (−1.96) At 6 months: WG: +1.79% (+0.1) SG: −18.9% (1) PG: −33.9% (−2.08)	ODI (0-100) At 3 months: WG: −9.4% (−3.35) SG: −22% (−7.14) PG: −27.3% (−9.06) At 6 months: WG: −6.9% (−2.47) SG: −6.2% (−2) PG: −16.7% (−5.53)	▪ Small sample size. ▪ High drop-out rate during the follow-up period.
Shnayderman and Katz-Leurer ^54^ 52 patients, Aged: 18-65 years	• Group 1 (WG): Received walking exercise on a treadmill (at 50% heart rate reserve, 40 min session, 2 sessions per week). • Group 2 (SG): specific low back strengthening exercises (2 times per week). • Duration: 6 weeks.	• Disability (ODI) • Measurements were done at baseline and after the intervention.	✓ Both groups showed significant improvements in all outcomes. ✓ No significant differences between groups.	Did not measure	Scale: ODI (0-100) After intervention (6 weeks): EG: −34.3% (−11.8) CG: −30.6% (−8.4)	▪ The participants were not classified and divided into groups according to sign and symptoms. ▪ Short study duration.
Murtezani *et al.* ^59^ 101 patients, Aged: 18-65 years	• EG: Received high-intensity aerobic exercise including treadmill walking, stair climbing, and stationary bicycling (at 50%-85% HRR, 30-50 mins sessions, three sessions per week). • CG: Passive modalities including interferential current, TENS, ultrasound, heat. (45 min session, three times per week) • Duration: 12 weeks.	• Pain intensity (VAS) • Disability (ODI) • Measurements were done at baseline and after the intervention.	✓ The experimental group showed significant improvements in all parameters. ✓ Improvements in the control group were not significant	Scale: VAS (0-10 cm) After intervention (12 weeks): EG: −66.7% (−4) CG: −1.6% (−0.1)	Scale: ODI (0-100) After intervention (12 weeks): EG: −49% (−15.2) CG: −0.3% (−0.1)	▪ No long-term follow-up was done. ▪ Not single or double-blinded.
Kell and Asmundson ^56^ 27 patients, Aged: >18 years	• RT group: Received periodized resistance training. • AT group: Received periodized progressive overload aerobic training (elliptical trainer and treadmill walking and jogging. 20-35 min session, 3 sessions per week). • CG: Maintained normal activity. • Duration: Total 14 weeks of exercise. (2 phases, 7 weeks per phase)	• Pain (VAS) • Disability (ODI) • Measurements were recorded at baseline, at 8 weeks, and at 16 weeks.	✓ RT groups – Significant improvement in pain, disability. ✓ AT group - More significant improvement in ODI compared to the control group but lesser than RT.	Scale: VAS (0-10 cm) At 8 weeks: RT: −27.8% (−1.5) AT: −5.9% (−4.9) At 16 weeks: RT: −38.9% (−2.1) AT: −5.9% (−0.3) CG: −2% (−0.1)	Scale: ODI (0-100) At 8 weeks: RT: −30.2% (−12.2) AT: −4.3% (−1.7) At 16 weeks: RT: −40.1% (−16.2) AT: −9.8% (−3.9) CG: −0.2% (−0.1)	▪ Small sample size.
Chatzitheodorou *et al.* ^57^ 20 patients, Aged: 25-65 years	• EG: High-intensity aerobic exercise (running on a treadmill at 60%-85% of HRR 30-50 mins session, 3 sessions per week). • CG: Received passive modalities including short-wave diathermy, ultrasound, laser, and electrotherapy without any PA. (45 min session). • Duration: 12 weeks.	• Pain intensity (MPQ) • Disability (RMDQ) • Measurements were done at baseline and after the intervention.	✓ Significant improvements in pain and disability were observed in the experimental group.	Scale: MPQ (0-78) After intervention (12 weeks): EG: −40.1% (−21.6) CG: −0.6% (−0.3)	Scale: RMDQ (0-24) After intervention (12 weeks): EG: −30.4% (−4.2) CG: −0.7% (−0.1)	▪ Small sample size. ▪ No long-term follow-up.

**Table 4.** Overview of included studies that evaluated the effectiveness of aerobic exercise combined with other intervention.

Study and subjects	Intervention	Measurements	Key outcomes	Mean change in percentage (absolute value change) pain	Mean change in percentage (absolute value change) disability	Key limitations
Suh *et al.* ^47^ 48 patients, Aged: >20 years	• FE group – Received flexibility exercise (stretching exercise for 30 mins). • WE group – Received Walking exercise. (fast walking on flat ground with abdominal bracing for 30 mins) • SE group – Received stabilization exercise (5 min warm-up and 25 mins of stabilization exercise according to patients exercise capacity). • SWE – stabilization and walking exercise (30 mins SE and 30 mins WE). • Duration: 6 weeks (30 to 60 minutes session, 5 sessions per week), and patients were advised to continue the exercise till the second follow-up at 12 weeks.	• Pain intensity during rest and physical activity (VAS). • Disability (ODI) • Measurements were done at baseline, within 2 weeks after the intervention, and at 6 weeks after the intervention.	✓ Pain intensity during physical activity was significantly decreased in all 4 groups. ✓ Exercise frequency was significantly increased in the SE and WE group. ✓ The endurance of supine, side-lying, and prone posture were significantly improved in the WE and SWE groups.	Scale: VAS (100 mm) Within 2 weeks after intervention (6 weeks): At rest: FE: −33.61% (−14.03) WE: −18.25% (−5.58) SE: −35.33% (−13.25) SWE: −38.9% (−11.67) On PA: FE: −45.26% (−31.16) WE: −38.7% (−23.07) SE: −48.19% (−33.25) SWE: −44.06% (−26.25) 6 weeks after intervention (12 weeks): At rest: FE: −22.47% (−9.42) WE: −34.6% (−10.58) SE: −40% (−15) SWE: −30.57% (−9.17) On PA: FE: −47.49% (−32.7) WE: −49.67% (−29.61) SE: −62.31% (−43) SWE: −50.64% (20.03)	Scale: ODI (0-100) Within 2 weeks after intervention (6 weeks): FE: −16.05% (−6.1) WE: −19.93% (−5.6) SE: −19.43% (−6.1) SWE: −18.09% (−5.5)	▪ Short study period ▪ No control groups.
Barni *et al.* ^50^ 22 patients, Aged: >18 years	• EG: Received 15 minutes of aerobic exercise (on the stationary bike at 65% HRR) and back school program. • CG: Received only back school program. • Duration: 5 weeks (2 sessions per week, per session, consisted of 90 minutes).	• Pain intensity (NRS) • Disability (RMDQ). • Measurements were recorded before and after the intervention.	✓ More significant reduction in NRS and RMDQ index in the experimental group than in the control group.	Scale: NRS (0-10 point) After intervention (5 weeks): EG: −27% (−1.85) CG: −13.14% (−0.87)	Scale: RMDQ (0-24) After intervention (5 weeks): EG: −25.58% (−2.75) CG: −10.3% (−1.12)	▪ No long-term follow-up. ▪ Small sample size. ▪ Short duration of intervention. ▪ Functional test was not performed.
Eadie *et al.* ^58^ 60 patients, Aged: 18-70 years	• Group 1 (WG): Received walking exercise (30 mins at moderate intensity, 5 days per week). (Pedometer was used to record the progress) • Group 2 (SG): Received supervised exercise class (back to fitness program, once per week). • Group 3 (PG): Received usual physiotherapy (advice, manual therapy, and exercise) • Duration: 8 weeks.	• Pain (NRS) • Disability (ODI) • Measurements were done baseline, 3 months, and 6 months.	✓ Greater pain intensity and disability improvements were observed in PG than WG and SG in all time points.	Scale: NRS (0-10) At 3 months: WG: −12.1% (−0.68) SG: −12.9% (−0.68) PG: −32% (−1.96) At 6 months: WG: +1.79% (+0.1) SG: −18.9% (1) PG: −33.9% (−2.08)	ODI (0-100) At 3 months: WG: −9.4% (−3.35) SG: −22% (−7.14) PG: −27.3% (−9.06) At 6 months: WG: −6.9% (−2.47) SG: −6.2% (−2) PG: −16.7% (−5.53)	▪ Small sample size. ▪ High drop-out rate during the follow-up period.
Krein *et al.* ^46^ 229 patients, Aged: >18 years	• EG: Received an uploading pedometer and had access to a website that provided automated walking goals, feedback, motivational messages, and social support through an e-community. • CG: Received an uploading pedometer but did not receive any walking goal and did not have access to the website (usual care group). • Duration: 12 months.	• Pain intensity (NRS) • Pain-related disability (RMDQ) • Measurements were done at baseline, 6 months, and 12 months.	✓ Greater improvement in RMDQ was observed in the experimental group at 6 months. ✓ Pain intensity improved in both groups at 6 months and 12 months; however, at 6 months, improvements were more significant.	Scale: NRS (0-10) At 6 months: EG: −21.7% (−1.3) CG: −14.8% (−0.9) At 12 months: EG: −10% (−0.6) CG: −8.2% (−0.5)	Scale: RMDQ (0-24) At 6 months: EG: −20.9% (−1.9) CG: −6.1% (−0.6)	▪ Patients were recruited from one medical center.
Marshall *et al.* ^52^ 64 patients, Aged: 18-50 years	• Group 1 (SEG): Received specific trunk exercise (35-40 min session, 3 sessions per week). • Group 2 (CEG): Received stationary cycling exercise (35-40 min session, 3 sessions per week). • Both groups attended exercise classes (50-60 min session, 3 times per week). • Duration: 8 weeks.	• Pain (VAS) • Disability (ODI) • Measurements were done at baseline, after the intervention, and at 6 months from the start of the intervention.	✓ Disability significantly lower in the SEG compared to CEG after the intervention. ✓ Pain decreased in both groups after the intervention, while SEG showed better improvement. ✓ Overall results suggested no long-term differences in outcomes between groups.	Scale: VAS (0-10 cm) After intervention (8 weeks): SEG: −52.8% (−1.9) CEG: −17.8% (−0.8) At 6 months: SEG: −44.4% (−1.6) CEG: −26.7% (−1.2)	Scale: ODI (0-100) After intervention (8 weeks): SEG: −40.9% (−10.4) CEG: −16.3% (−3.9) At 6 months: SEG: −40.9% (−10.4) CEG: −24.6% (−5.9)	▪ Severely impaired patients were not recruited. ▪ No blind exercise supervision.
McDonough *et al.* ^53^ 57 patients, Aged: 43-53 years	• EG: Pedometer-driven walking program + education. • CG: Received only education or advice. • Duration: 8 weeks.	• Pain intensity (NRS 0-10) • Functional disability (ODI 0-100) • Measurements were recorded at baseline, at week 9 (immediately after intervention), and at 6 months.	✓ Pain intensity improved in both groups; however, EG showed greater improvement. ✓ EG showed a better outcome in disability.	Scale: NRS (0-10) After intervention (9 weeks): EG: −16.7% (−0.9) CG: −15.2% (−0.7) At 6 months: EG: −29.6% (−1.6) CG: −10.9% (−0.5)	Scale: ODI (0-100) After intervention (9 weeks): EG: −17.2% (−5.5) CG: −3.3% (−0.9) At 6 months: EG: −25.7% (−8.2) CG: −5.5% (−1.5)	▪ Relatively small sample size.
Chan *et al.* ^55^ 46 patients, Aged: >18 years	• EG: Received aerobic exercise including treadmill walking or running, stepping, cycling exercises; selected by patient's preference (20 min session, 3 times a week at 40%-60% HRR, gradually progressed up to 85% at a 5% increment each weak) + conventional physiotherapy • Control group: Only conventional physiotherapy • Duration: 8 weeks.	• Pain intensity (VAS) • Functional disability (ALBPS) • Measurements were done at baseline, 8 weeks, and 12 months from the start of the intervention.	✓ Significant improvement in pain and disability in both groups at 8 weeks. ✓ Improvement in disability sustained in both groups at 12 months. ✓ No significant differences were observed between groups. ✓ No significant difference in LBP relapse at 12 months between two groups.	Scale: VAS (0-100 mm) After intervention (8 weeks): EG: −47.1% (−28) CG: −42% (−25)	Scale: ALBPS (0-100) After intervention (8 weeks): EG: −34% (−9.8) CG: −32.5% (−10) At 12 months: EG: −36.1% (−10.4) CG: −22.1% (−6.8)	▪ The poor baseline fitness level of patients. ▪ Short duration of intervention. ▪ Relatively small sample size.
Koldas *et al.* ^48^ 60 patients, Aged: >25 years	• Group 1 (AHE): Aerobic exercise (40-50 min of exercise on a treadmill at 65-70% HRR, 3 times a week for 6 weeks) + home exercise. • Group 2 (PT): Physical therapy (hot pack, ultrasound, and TENS). • Group 3 (HE): Home exercise only (basic flexion, extension, mobilization, and stretching with 15-20 repetitions, once a day for 6 weeks). • Duration: 6 weeks.	• Pain intensity (VAS) • Disability (RMDQ) • Measurements were done at baseline and at the end of the intervention. Follow-up was done at 1 month after the intervention.	✓ Pain reduced significantly in all three groups after the treatment and at 1-month follow-up. ✓ Disability improved significantly in PT at follow-up. ✓ No significant differences in pain intensity, disability were observed between three groups after the treatment and at follow-up.	Scale: VAS (0-100 mm) After intervention (6 weeks): AHE: −39.6% (−22.15) PT: −36.5% (−22.3) HE: −28.6% (−16) At 1 month follow up: AHE: −38.8% (−22.95) PT: −53% (−32.4) HE: −40% (−22.4)	Scale: RMDQ (0-24) After intervention (6 weeks): AHE: −25.2% (−3) PT: −25.2% (−3) HE: 0% (0) At 1 month follow up: AHE: −22.7% (−2.7) PT: −30.3% (−3.6) HE: −2.2% (−0.3)	▪ No control groups. ▪ Relatively small sample size. ▪ Short duration of intervention.

## Discussion

Walking is a highly cost-effective AE that is regularly advised to patients with CLBP. ^44^^,^^60^^,^^61^ This exercise is easy to perform and does not require any particular skill or facilities. ^60^^,^^62^ It is regarded as one of the safest exercises because of its low injury rate. ^60^^,^^62^ Also, walking enhances cardio-respiratory capacity, maximum oxygen uptake, and prevents LBP by increasing the isometric endurance of muscles. ^63^^,^^64^

In this review, three good-quality studies (PEDro score ≥ 7) compared walking alone exercise to other interventions. ^44^^,^^49^^,^^54^ Studies showed that walking alone exercise effectively reduced pain and disability in CLBP patients. ^44^^,^^49^^,^^54^ However, there was no evidence that walking alone exercise was superior to other interventions. Similar outcomes were reported in three different reviews, and they concluded that walking exercise was as effective as other interventions. ^30^^–^^32^ Moreover, in this review, walking alone exercise was not effective in attaining clinically significant improvements (≥ 30%) in pain intensity and disability on most occasions. ^44^^,^^49^^,^^54^ In previous studies, exercise alone therapy was not effective to make clinically significant changes (≥ 30%) in CLBP patients; therefore, it was suggested to apply combined treatment in clinical trials. ^22^^,^^65^ Lawford *et al.* ^32^ also asserted in their review that combined walking exercise was more effective than walking exercise alone.

In this review, three studies (fair to good quality) examined the effectiveness of walking exercise combined with other interventions. ^47^^,^^48^^,^^51^ Results indicated that walking exercise combined with other interventions effectively reduced pain intensity in CLBP patients, and the improvements were clinically meaningful (≥30%). ^47^^,^^48^^,^^51^ Besides, disability improvements were statistically significant in all three studies. ^47^^,^^48^^,^^51^ However, these enhancements (<30%) were not remarkable enough to be clinically significant. It was claimed that to get better improvements in any intervention, patients should be trained for a sufficiently longer period. ^51^ The duration of these studies was ≤ 8 weeks; hence, the duration could be too small to make any clinically significant improvement in disability. Moreover, exercise effectiveness can be improved by adjusting the intensity and duration of exercise according to patients' capacity. ^66^ Research showed that atrophic changes in lumbar paraspinal muscles are common in CLBP patients, which could decrease patients' ability to do prolonged exercise. ^67^^,^^68^ One of the included studies also showed that it was difficult for the patients to continue 60 minutes of exercise. ^47^ Hence the authors advised selecting an exercise program with a duration of about 30 minutes. ^47^

Recently, internet-based programs are used to promote healthy behaviors. ^69^^–^^71^ Some studies were carried out to see if pedometer-driven walking exercise could benefit CLBP patients' conditions. ^46^^,^^53^^,^^58^ In this review, the findings of three included RCTs (PEDro score = 8) indicated that walking exercise in conjunction with advice, education, or support effectively improved CLBP patients' conditions. ^46^^,^^53^^,^^58^ However, there was no clear evidence that using a pedometer had any extra benefits. A pedometer can guide a patient to track their progress, but further research is needed to recommend pedometer as rehabilitation tools for CLBP patients.

Stationary cycling exercise is another form of AE which is regarded as one of the most effective exercises to improve muscular coordination. ^50^^,^^72^ Three fair to good quality RCTs were included in this review that examined the effectiveness of stationary cycling exercise, and outcomes demonstrated that stationary cycling exercise was as effective as walking exercise, and stationary cycling exercise was not inferior to other interventions. ^45^^,^^50^^,^^52^ In addition, Chatzitheodorou *et al.* ^56^ showed that high-intensity AE, including running, was adequate to improve pain and disability in CLBP patients significantly. In another study, Murtezani *et al.* ^59^ demonstrated that a combination of different AE effectively improved conditions of CLBP patients. These included studies also showed the possible influence of exercise frequency and duration of intervention on outcomes. The study with the higher frequency of exercise (five days/week) ^50^ and longer duration (12 weeks) of intervention ^57^^,^^59^ showed clinically significant (≥ 30%) changes in both pain and disability scores. ^50^ The American College of Sports Medicine recommended that physical activity should be performed for 30 minutes at moderate intensity with a frequency of five days/week. ^73^^–^^75^ Therefore, the exercise frequency and duration of intervention could be the keys to obtain clinically significant improvements in pain intensity and disability in CLBP patients. However, future studies with a larger sample size and long-term follow-up are required to justify it.

Individually tailored and supervised exercise programs were suggested by Hayden *et al.* ^76^ in their meta-analysis. In this review, one good quality study (PEDro = 7) showed that individually designed and supervised aerobic exercise effectively made clinically significant improvements in patients with CLBP. ^55^ In addition, one good quality study (PEDro = 7) was included in this review that examined the impact of the environment on the effectiveness of aerobic exercise. ^43^ Patients were randomly allocated and received the same exercise either on land or in water, and results indicated that the environment did not influence the outcomes. ^43^ However, more studies with a large sample size and long-term follow-up are required for further evidence.

Only one included study in this review did an experiment to observe the effectiveness of periodized progressive overload training. ^56^ Results showed that periodized progressive overload aerobic training failed to improve pain intensity and disability in CLBP patients. ^56^ However, the study was regarded as fair quality (PEDro score 5); therefore, a good quality study with a larger sample size is warranted to conclude the effectiveness of periodized progressive overload aerobic training on CLBP patients.

## Strengths and limitations of the review

There are several strengths of this review. A highly sensitive search of different databases was performed to find the relevant studies. A total of 17 RCTs were included in this review that involved an adequate sample size of 1146 participants after applying strict inclusion and exclusion criteria. Moreover, PEDro scoring was done to assess quality and risk of bias, and all included studies scored ≥ 4 to be considered fair to good quality.

In contrast, in this review, only improvements in pain intensity and disability were evaluated, which was considered a major limitation. Other outcomes, including fear-avoidance beliefs, mental and physical health, quality of life, and cost-effectiveness of interventions, were missed. In addition, studies published other than the English language were excluded in this review. Therefore, it is highly recommended for further review to include studies in different languages and from lesser-known databases.

## Conclusion

Overall, this review showed that AE effectively reduced pain intensity and functional disability in CLBP patients. It also demonstrates the appositeness of using AE as an intervention in future studies. Findings of most of the included studies demonstrated that patients gained statistically significant pain intensity and disability improvements. Results indicated that exercise should be done under supervision at a minimum frequency of 5 days/week, for at least 12 weeks, and in combination with other interventions including education, physiotherapy, home exercise, or other forms of exercise to get clinically significant outcomes. Future studies should emphasize training patients at the optimum frequency, intensity, and duration so that the participants can achieve clinically meaningful improvements.

## Data availability

## Underlying data

All data underlying the results are available as part of the article and no additional source data are required.

## Reporting guidelines

Mendeley Data: PRISMA checklist for ‘The effectiveness of different aerobic exercises to improve pain intensity and disability in chronic low back pain patients: A systemic review.’

https://doi.org/10.17632/79vnhtfh85.2 ^77^

This project contains the following data:

• PRISMA checklist.doc

Data are available under the terms of the Creative Commons Attribution 4.0 International license (CC-BY 4.0).
