# Supplementary material for: The effectiveness of different aerobic exercises to improve pain intensity and disability in chronic low back pain patients: a systematic review
Source: F1000Res. 2023 Jul 18;11:136. Originally published 2022 Feb 2. [Version 2] doi: 10.12688/f1000research.75440.2 (PMC10579857; doi:10.12688/f1000research.75440.2)
Supplement: Peer review history

# Reviewer report: Reviewer response for version 2

**DOI:** 10.5256/f1000research.152869.r198793

**Published:** 2023-10-16

**Authors:** G Shankar Ganesh

**Affiliations:** 1 Composite Regional Centre for Skill Development, Rehabilitation, and Empowerment of Persons with Disabilities, Lucknow, Uttar Pradesh, India

Dear authors,

 Numerous SRs have assessed the efficacy of various exercises in enhancing the outcomes of patients with CLBP. The authors intended to evaluate the evidence for the efficacy of aerobic exercises (mode and intensity) in enhancing clinical outcomes related to pain and disability. The primary limitation is the lack of a consistent comparison group, which, if present, should be stated explicitly by the authors. In addition, the results should be interpreted in light of the fact that there are not many additional advantages of aerobic exercises compared to the groups receiving alternative interventions. The results should be categorised and presented in accordance with the declared objectives of the study. In its present form, the manuscript does not provide a conclusive summary of these lines. In accordance, the manuscript must be revised.

 Other points to consider while revising  are:

1. Reframe the sentence: Aerobic exercise (AE) is one of the most recommended and widely used CLBP patients' interventions.
2. The explanation for an SR regarding why this objective must be reviewed is not supported.
3. 'However, neither of these reviews included a study published after 2013'. Please check the veracity of this statement, as the authors of the SRs have stated that they considered literature searches until the year of study.
4. Please state if the trial was prospectively registered with PROSPERO. If yes, provide the ID.
5. Please state what MESH words were used and explain the search methodology with respect to Boolean operators and free text (if used).
6. Please describe your objective using the PICO format. The research should specify the intervention with which the interventions (aerobic exercise) were compared. The agent of comparison is not specified. However, it has to be reminded here that the study hypothesis is different.
7. The contents of Table 1 are already elaborated upon in the text. This must be removed. Likewise, since the PEDro database is well-known, the authors may wish to remove the section describing its components.
8. What heterogeneity was observed in the measurement of outcomes?
9. Due to the fact that the control groups were given distinct exercises and the study's objectives were not met, the results cannot be considered valid. The ideal study would have broken the results of aerobic exercises into modes (different categories of exercises), durations (short, medium, and long), and intensities (moderate or high).
10. The results do not support the study's conclusions.

Regards.

Are the rationale for, and objectives of, the Systematic Review clearly stated?

No

Is the statistical analysis and its interpretation appropriate?

Not applicable

Are sufficient details of the methods and analysis provided to allow replication by others?

No

Are the conclusions drawn adequately supported by the results presented in the review?

No

Reviewer Expertise:

pain

I confirm that I have read this submission and believe that I have an appropriate level of expertise to confirm that it is of an acceptable scientific standard, however I have significant reservations, as outlined above.

# Reviewer report: Reviewer response for version 1

**DOI:** 10.5256/f1000research.79315.r126016

**Published:** 2022-03-11

**Authors:** Moshiur Rahman Khasru

**Affiliations:** 1 Department of Physical Medicine and Rehabilitaion, Bangabandhu Sheikh Mujib Medical University, Dhaka, Bangladesh

Thanks for providing an opportunity to review this systematic review.

 I have a few comments:

- Information regarding inclusion or exclusion of Inflammatory back pain should be stated clearly in this review.
- Was this review registered to any registration authority?
- It would be great if meta-analysis could be addressed - factors directing authors not to go for meta-analysis could be stated in the methods.

However, this manuscript can be accepted for publication.

Are the rationale for, and objectives of, the Systematic Review clearly stated?

Yes

Is the statistical analysis and its interpretation appropriate?

Yes

Are sufficient details of the methods and analysis provided to allow replication by others?

Yes

Are the conclusions drawn adequately supported by the results presented in the review?

Yes

Reviewer Expertise:

Rheumatic disease, Stem cells, and pain

I confirm that I have read this submission and believe that I have an appropriate level of expertise to confirm that it is of an acceptable scientific standard.

# Reviewer report: Reviewer response for version 1

**DOI:** 10.5256/f1000research.79315.r121997

**Published:** 2022-02-04

**Authors:** Navid Moghadam

**Affiliations:** 1 Sports Medicine Research Center, Neuroscience Institute, Tehran University of Medical Sciences, Tehran, Iran

Thanks for the opportunity to review.

 It is an update to previous systematic reviews but I have some concerns before approval: 

 1. The search query is not disclosed; it should at least be contained as a supplement.

 2. The reason for not doing a meta-analysis is not described, is this due to issues of heterogeneity?

 3. The main outcome measure is not described in terms of the questionnaire.

Are the rationale for, and objectives of, the Systematic Review clearly stated?

Yes

Is the statistical analysis and its interpretation appropriate?

Partly

Are sufficient details of the methods and analysis provided to allow replication by others?

Partly

Are the conclusions drawn adequately supported by the results presented in the review?

Partly

Reviewer Expertise:

Low back pain

I confirm that I have read this submission and believe that I have an appropriate level of expertise to confirm that it is of an acceptable scientific standard, however I have significant reservations, as outlined above.
